# Joint Multi-Fiber NODDI Parameter Estimation and Tractography Using the Unscented Information Filter

**DOI:** 10.3389/fnins.2016.00166

**Published:** 2016-04-20

**Authors:** Chinthala P. Reddy, Yogesh Rathi

**Affiliations:** ^1^Data Analytics, Walmart ISDBangalore, India; ^2^Psychiatry Neuroimaging Laboratory, Harvard Medical SchoolBoston, MA, USA

**Keywords:** diffusion-weighted MRI, tractography, filtering, NODDI, Neurite Orientation dispersion, Information Filter

## Abstract

Tracing white matter fiber bundles is an integral part of analyzing brain connectivity. An accurate estimate of the underlying tissue parameters is also paramount in several neuroscience applications. In this work, we propose to use a joint fiber model estimation and tractography algorithm that uses the NODDI (neurite orientation dispersion diffusion imaging) model to estimate fiber orientation dispersion consistently and smoothly along the fiber tracts along with estimating the intracellular and extracellular volume fractions from the diffusion signal. While the NODDI model has been used in earlier works to estimate the microstructural parameters at each voxel independently, for the first time, we propose to integrate it into a tractography framework. We extend this framework to estimate the NODDI parameters for two crossing fibers, which is imperative to trace fiber bundles through crossings as well as to estimate the microstructural parameters for each fiber bundle separately. We propose to use the unscented information filter (UIF) to accurately estimate the model parameters and perform tractography. The proposed approach has significant computational performance improvements as well as numerical robustness over the unscented Kalman filter (UKF). Our method not only estimates the confidence in the estimated parameters via the covariance matrix, but also provides the Fisher-information matrix of the state variables (model parameters), which can be quite useful to measure model complexity. Results from *in-vivo* human brain data sets demonstrate the ability of our algorithm to trace through crossing fiber regions, while estimating orientation dispersion and other biophysical model parameters in a consistent manner along the tracts.

## 1. Introduction

Diffusion MRI (dMRI) is a non-invasive technique to study the microstructure of brain tissue. However, we need a mathematical model to interpret the diffusion weighted signal to study the microstructure of white matter fibers. Broadly, such models fall into two categories: parametric and nonparametric. The simplest parametric model is the diffusion tensor model, which describes a Gaussian estimate of the strength and diffusion orientation at each voxel (Basser et al., 1994; Alexander et al., 2002). While robust, this model can be inadequate in cases of mixed fiber presence or more complex orientations (Frank, 2002). To handle more complex diffusion patterns, parametric models have been introduced including mixtures of tensors and directional functions (Alexander et al., 2001; Anderson, 2005; Kreher et al., 2005; Parker and Alexander, 2005; Peled et al., 2006; Kaden et al., 2007; Zhang et al., 2007; Rathi et al., 2008). Several techniques attempt to reconstruct pathways based on these models. In this case, tractography is done by following the principal diffusion direction(s).

With multifiber models, care must be taken in fitting the model parameters to the recorded signal consistently, such that, the correlation in diffusion along the tract is accounted for while estimating the parameters (Malcolm et al., 2009a). As demonstrated in this paper, estimation based on information from previous estimates aids in this process.

Nonparametric techniques, unlike estimating a fixed number of fibers in parametric models, estimate an oriented distribution function (ODF) describing an arbitrary configuration of fibers. For this estimation, Tuch (2004) introduced Q-ball imaging to numerically compute the ODF via the Funk-Radon transform, and later, spherical harmonics were used to simplify the computation with an analytic form (Anderson, 2005; Hess et al., 2006; Ozarslan et al., 2006; Descoteaux et al., 2007). Another method to estimate the fiber ODF is to assume a model for the signal response of a single-fiber and use spherical deconvolution (Jansons and Alexander, 2003; Tournier et al., 2004; Jian and Vemuri, 2007; Kaden et al., 2007) to obtain a much sharper orientation profile. A good review of both parametric and nonparametric models and diffusion MRI in general can be found in Assemlal et al. (2011).

Recently, a model of diffusion was proposed by Zhang et al. (2012) called Neurite Orientation Dispersion Diffusion Imaging or NODDI, which accounted for the dispersion in orientation of the axonal fibers. In this work, the authors proposed an algorithm to estimate the parameters of the NODDI model assuming the existence of a single fiber pathway at each voxel. However, it is well known that most of the white matter tissue in the brain has crossing fibers (Tuch et al., 2002), which must be taken into account for proper analysis of the estimated microstructural parameters. Further, the correlation in diffusion (and consequently the estimated NODDI parameters) is not taken into account in the work of Zhang et al. (2012). Thus, estimating the orientation dispersion in the presence of multiple fiber crossing would be quite useful in analyzing the geometrical structure of white matter in healthy as well as diseased subjects.

### 1.1. Our contributions

In this work, we propose to use the unscented information filter (UIF) based framework to perform joint NODDI parameter estimation and tractography. Earlier works have used the unscented Kalman filter (UKF) with a parametric multi-tensor model or a non-parametric spherical harmonic representation to do simultaneous model estimation and tractography (Malcolm et al., 2010; Baumgartner et al., 2012). The UIF has several advantages over the UKF as has been noted in Lee (2008), namely, it propagates the Fisher-information matrix as opposed to the state-covariance matrix leading to a significant reduction in computational load, while providing a more robust estimation of the estimated parameters. Further, the Fisher-information is calculated for each of the model parameters along the tract, providing the variance in the estimation of the model parameters.

Existing methods either use a region-of-interest (ROI) or an atlas within which the NODDI parameters are estimated and analyzed or follow the principal orientation to perform tractography. Thus, the fit is performed independently at each voxel disregarding the correlation of diffusion along the fiber path. In the UIF framework, we perform model estimation and tractography simultaneously to trace fiber tracts. The methodology assumes a “time varying" Gaussian distribution of the model parameters as the algorithm moves from one location to the next. This allows for consistent estimation of the NODDI parameters, while allowing for smooth estimation of the fiber tracts. In the current work, we demonstrate the performance of our method in the case of single fiber and 2-fiber NODDI model. To the best of our knowledge, this is a first extension of the NODDI model to the multi-fiber case within a tractography framework. Thus, the ability to trace multiple crossing fibers while robustly estimating the NODDI model parameters is one of the major contributions of this work. We demonstrate our technique on *in-vivo* human data set from the human connectome project (HCP) data set. We expect, that the proposed method will be quite useful to study the tissue microstructure in several disorders.

## 2. Approach

The main idea of our approach is to trace the local fiber orientations employing the three compartment NODDI model (intra-cellular, extra-cellular, and isotropic free-water) and using the estimation at previous positions to guide estimation at the current position. In a recursive fashion, the information filter estimates the model at the current position, moves a step in the most consistent direction, and then begins estimation again. In this case, the model parameters, which form the state of the filter, are assumed to have a Gaussian distribution, whose mean and covariance change at each step. Thus, the space of possible solutions (among the infinitely many possibilities) is greatly reduced leading to better accuracy in resolving individual orientations and yielding inherently smooth tracts despite the presence of noise and uncertainty. This is in complete contrast to existing methods, where the model parameters at each voxel are estimated from an arbitrary initialization or a large number of random initializations to finally obtain a solution that best fits the data. This could potentially lead to vastly different solution even in neighboring voxels since the space of possible solutions is large. In the proposed method, since each iteration begins with a near-optimal solution provided by the previous estimation, and since the step size is kept to be very small, the UIF quickly converges to the optimal solution and many local minima are naturally avoided.

### 2.1. Modeling local fiber orientations

The three compartment NODDI model consists of an intra-cellular, an extra-cellular and an isotropic compartment (Zhang et al., 2012; Daducci et al., 2015). The normalized signal *E* can be written as:

(1) $$\begin{array}{l} E=\left(1-V_{\;iso}\right)\left(V_{\;ic}E_{\;ic}+\left(1-V_{\;ic}\right)E_{\;ec}\right)+V_{\;iso}E_{\;iso}, \end{array}$$

where *E*_*ic*_ and *V*_*ic*_ are the normalized signal and volume fractions of the intra-cellular compartment; *E*_*ec*_ is the normalized signal corresponding to the extra-cellular compartment; and *E*_*iso*_ and *V*_*iso*_ are the normalized signal and volume fractions of the isotropic compartment. The space bounded by the membrane of neurites is called the intra-cellular compartment. In this paper, we adopt a model where neurites are modeled as a set of cylinders of zero radius capturing the unrestricted nature of diffusion along the neurites and restricted diffusion perpendicular to them, i.e.,

$$\begin{array}{l} E_{ic}(u)=\int_{S^{2}}f(n)e^{-bd_{∥}{\left(u\cdot n\right)}^{2}}dn, \end{array}$$

where b is the *b*-value, **u** is the gradient direction; *f*(**n**) is the orientation dispersion function around vector **n**; and *d*_∥_ is intrinsic diffusivity along the cylinder. As in Zhang et al. (2012), the watson distribution is used to model the orientation dispersion function *f* : 𝕊^2^ → ℝ, *f*(**n**) = *M*(1/2, 3/3, κ)^−1^*e*^κ(**m**·**n**)^^2^, where *m* is the mean orientation about which the dispersion is symmetric and κ is the concentration parameter, which determines the extent of orientation dispersion along the mean orientation; and *M* is the confluent hypergeometric function. The orientation dispersion index is given by $OD=\frac{2}{\pi }\arctan\frac{1}{\kappa }$.

The space around the neurites is called the extra-cellular compartment. In this compartment, water diffusion is assumed to be hindered and is modeled as a Gaussian anisotropic tensor, with

$$\log E_{ec}(u)=-bu^{T}\left(\int_{S^{2}}f(n)D(n)dn\right)u,$$

where *D*(**n**) is a cylindrically symmetric diffusion tensor with principal orientation **n**, and *d*_∥_ and *d*_⊥_ are the coefficients of diffusion parallel and perpendicular to **n**. Both these parameters can expressed in terms of the intra-cellular fraction *V*_*ic*_ and the concentration parameter κ (see Zhang et al., 2012 for more details). The isotropic compartment is modeled with an isotropic Gaussian diffusion with diffusivity set to *d*_*iso*_.

### 2.2. Estimating the NODDI parameters

As described in Zhang et al. (2012), we fix the following parameters within the NODDI model: $d_{∥}=1.7\times 10^{-3}mm^{2}s^{-1}$ and $d_{iso}=3\times 10^{-3}mm^{2}s^{-1}$. Consequently, for a single fiber NODDI model, the free parameters are: *V*_*ic*_, κ, **m** and *V*_*iso*_. For the two-fiber NODDI model, we used the following formulation:

(2) $$\begin{array}{l} E=(1-V_{iso})(0.5\{V_{ic1}E_{ic1}+(1-V_{ic1})E_{ec1}\}\\ \;+0.5\{V_{ic2}E_{ic2}+(1-V_{ic2})E_{ec2}\})+V_{iso}E_{iso}, \end{array}$$

In this case, the free parameters to be estimated are: *V*_*ic*1_, κ_1_, **m_1_**, *V*_*ic*2_, κ_2_, **m_2_**, and *V*_*iso*_. Given the measured signal at a particular voxel, we want to estimate the above model parameters that best explain the signal. We propose to achieve this using the unscented information filter (UIF), which is a recursive non-linear least squares estimator, giving the maximum likelihood estimate of the model parameters. Further, the Fisher information computed by the UIF provides a lower bound on the precision with which the model parameters can be estimated given the data. This statistic can be summarized into a single number as the estimated uncertainity at each point, by computing the matrix norm of the estimated covariance matrix. This uncertainity measure can be quite useful in removing unlikely fibers (with high uncertainity) from the tractography obtained. Note that, such information is typically not available using other model estimation methods.

As in streamline tractography, we treat the fiber as the trajectory of a particle which we trace out. At each step, we examine the measured signal at that position, use that measurement to update our model parameters within the filter, and propagate forward in the most consistent direction. To use a state-space formulation for estimating the model parameters, we need the following application-specific definition of four filter components:

1. The system state (**x**): which are the model parameters in our case,
2. The state transition function (*s*): how the model changes as we trace the fiber,
3. The observation function (*h*): how the signal appears given a particular model state,
4. The measurement (**y**): the actual signal obtained from the scanner.

For our state (**x**), we directly use the parameters of the NODDI model (in the case of 2-fibers):

(3) $$\begin{array}{l} \text{x}=\left[V_{ic1}\kappa_{1}{\text{m}}_{1}V_{ic2}\kappa_{2}{\text{m}}_{2}V_{iso}\right]. \end{array}$$

For the state transition function *s*, we assume identity dynamics since the local fiber configuration does not undergo drastic change as it moves from one location to the next, when the step size is kept very small. The predicted signal is computed using: **y** = *h*[**x**] given by Equations (1) or (2) depending on the model used (1 fiber or 2-fiber NODDI) and our actual measurement is the actual signal interpolated directly on the diffusion weighted images at the current position.

It is important to note that we chose the unscented Information filter for its low computational complexity compared to the UKF. In the UKF formulation, one not only estimates the state, but also the state covariance matrix *P*, whose dimension is *k* × *k*, where *k* is the number of diffusion weighted gradients. At each step, the covariance matrix *P* is updated, which involves inversion of a large matrix that depends on *k*. This makes the filter computationally very expensive, especially in cases like the human connectome data, which has about 270 measurements. In contrast, in the UIF filter, only the Fisher-Information matrix is computed and propagated, which involves inversion of a matrix whose maximal dimension is substantially small, i.e., the length of the state vector. Thus, in the case of the two-fiber NODDI model, one only needs to invert the information matrix of size 11 × 11. This significantly increases the computational speed of the algorithm.

We use the unscented information filter with constraints, where the final solution is projected onto the physiological range of the parameters using a quadratic programming problem. For example, *V*_*iso*_, *V*_*ic*_ are constrained to lie between [0, 1], while κ is always set to be positive. For a more thorough treatment of the UKF and UIF filters with constraints (see Lee, 2008; Malcolm et al., 2009b, 2010). In Appendix, we describe the mathematical equations for the prediction and measurement update steps of the UIF filter.

### 2.3. The tractography algorithm

The UIF filter provides the maximum a-posteriori estimate of the model parameters, given the signal at each location. We embed this into a tractography framework, as described in detail in Malcolm et al. (2010). Briefly, we begin by initializing the UIF filter at each seed point by using the coarse-grid search method as described in Zhang et al. (2012). Subsequently, we run the UIF filter at each seed point, which provides an estimate of the model parameters, including the principal orientation(s) of the fiber bundle. A small step (using a fixed pre-determined step size) is taken along the direction of the principal fiber orientation that is most consistent as compared to the previous estimate. At sub-voxel locations, the signal is interpolated using an isotropic Gaussian kernel with its width (variance) given by the smallest voxel length in any direction. This interpolated signal is then used as the acquired measurement (*y*), within the UIF filter to estimate the model parameters as well as the Fisher information and the covariance matrix. In a loop, the fiber is then traced until a termination criteria is reached. In the present case, we used generalized fraction anisotropy (GFA) threshold of 0.08 and κ of 0.06 as the stopping criteria.

## 3. Experiments

We tested the proposed algorithm by tracing several fiber bundles from the *in-vivo* human data set obtained from HCP (Van Essen et al., 2013). All *b*-values of {1000, 2000, 3000}*s*/*mm*^2^ were used to perform whole brain tractography using single fiber and two-fiber NODDI models.

On a 2.4 GHz processor with 16 cores, the UKF filter (with 2-fiber NODDI model) required about 72 hours of computational time for a single subject whole-brain tractography with 1 seed per voxel on the HCP data set with 270 gradient directions. With the same set of parameters, the UIF-based whole brain tractography required about 30 hours, an improvement by more than a factor of 2.

Whole brain tractography was performed using with the following parameter settings: expected rate of change of orientation *q*_*m*_ = 0.002, rate of change of κ (dispersion), *q*_κ_ = 0.015 and expected rate of change of intracellular and isotropic volume fraction was set to *q*_*ic*_ = *q*_*iso*_ = 0.0007. The parameter *r*_*s*_ is akin to a regularization parameter that should be set based on the expected noise level in the data. In the case of HCP data, we set it to 0.02, based on the noise level in the data. Thus, for higher noise in the data, *r*_*s*_ should be increased, which implicitly implies that the algorithm will trust the model more than the data. On the other hand, for high SNR data, *r*_*s*_ should be reduced to allow the tractography method to trust the data more than the expected model. For all the experiments, we set the step length parameter to 0.5 mm.

**Figure 1:**
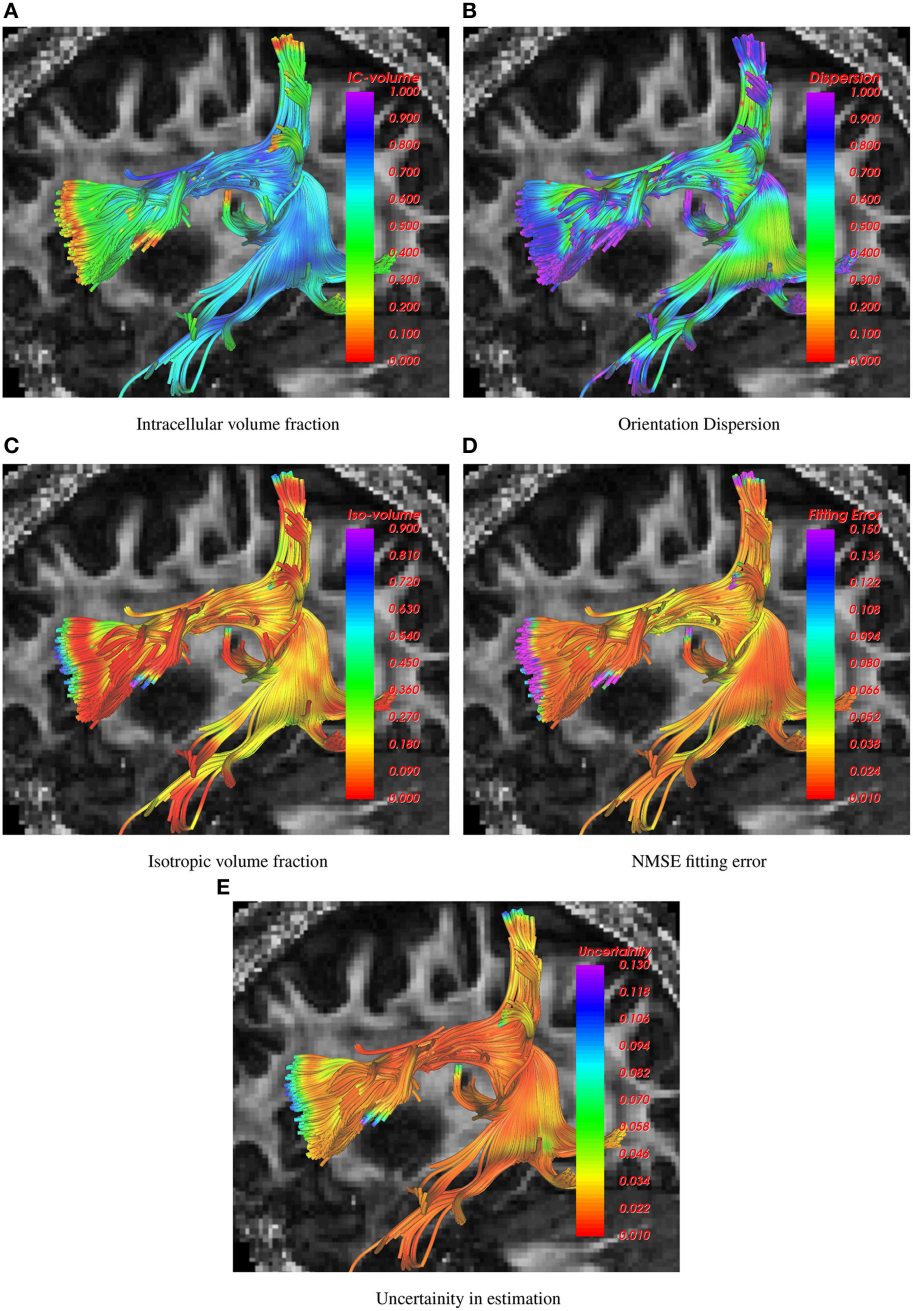
**Visualization of intracelluar volume fraction, fiber dispersion, isotropic volume fraction, data fitting error and uncertainity in parameter estimation using 1-fiber noddi model in the arcuate fasciculus**. The background slice is the single tensor FA map.

The following figures show the traced fiber bundles extracted using the white matter query language (WMQL) (Wassermann et al., 2013). In particular, Figure 1 shows the arcuate fasciculus traced using the 1-fiber NODDI model. Estimates of several diffusion measures of interest, such as, intracellular volume fraction, orientation dispersion, isotropic volume fraction, normalized mean squared error (NMSE) in fitting the data and uncertainity in the estimated parameters are shown along the tract with the standard single diffusion tensor based FA map shown in the background.

**Figure 2:**
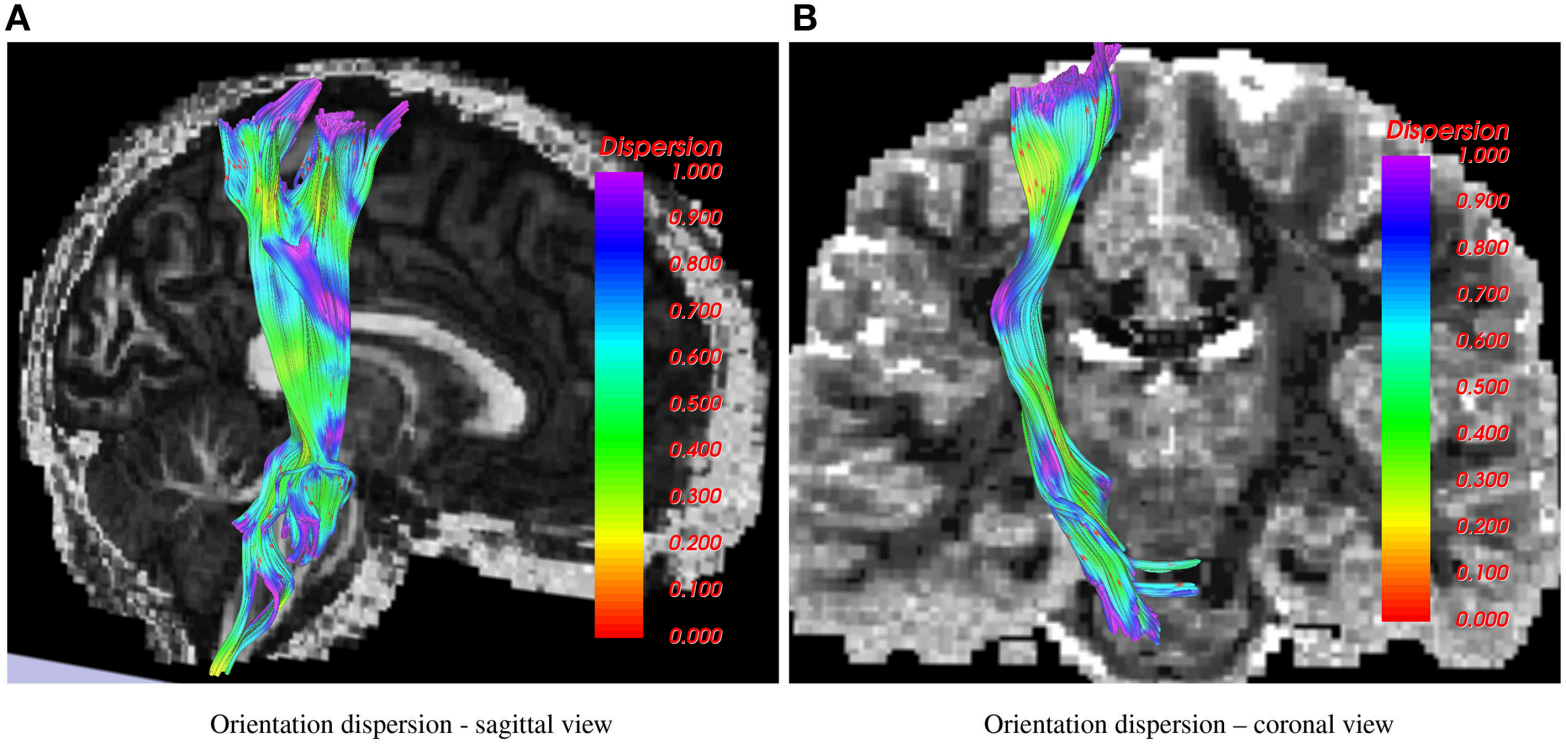
**Corticospinal tract showing orientation dispersion from two different views**. In the sagittal view, the background is FA, while in the coronal view the 1-fiber NODDI dispersion map (Daducci et al., 2015) is shown in the background.

In Figure 2, the corticospinal tract is shown as traced using the 1-fiber NODDI model. In the coronal view, the background is a slice of fiber orientation obtained using the method in Daducci et al. (2015). Note the similarity in the obtained measurements using both the methods, i.e., high dispersion in the CSF and gray matter areas and low in the deep white matter regions.

**Figure 3:**
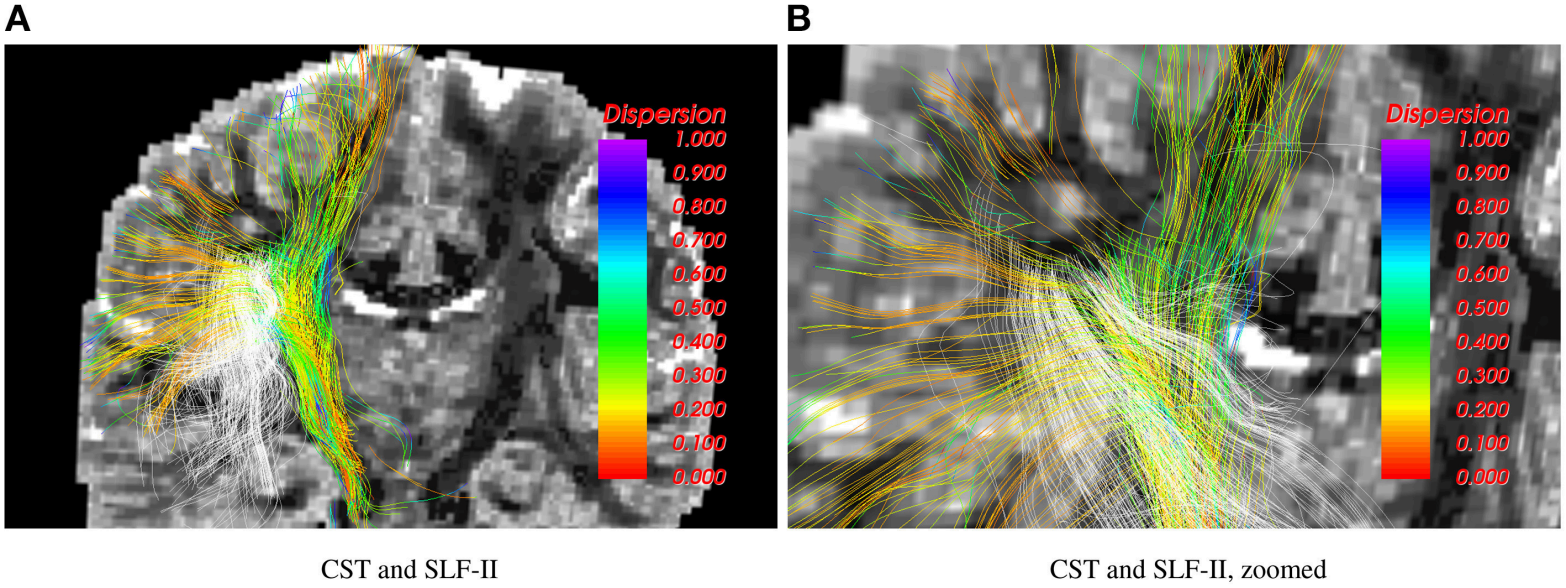
**Corticospinal tract (CST) and SLF-II traced using the 2-fiber NODDI model**. Intersecting fibers are seen in the centrum-semiovale region.

Figure 3 shows results for the 2-fiber NODDI model obtained using the UIF filter. The cortico-spinal tract (shown in color) and the superior-longitudinal fasiculus II (SLF-II) intersect in the centrum-semiovale region. As can be seen, the proposed algorithm is able to trace fibers through crossing regions, which is not possible using the 1-fiber NODDI model. Also, the lateral fibers of the corticospinal tract (CST) that go to the hand and face area are missing in the 1-fiber NODDI model, but can be nicely traced using the 2-fiber NODDI model.

**Figure 4:**
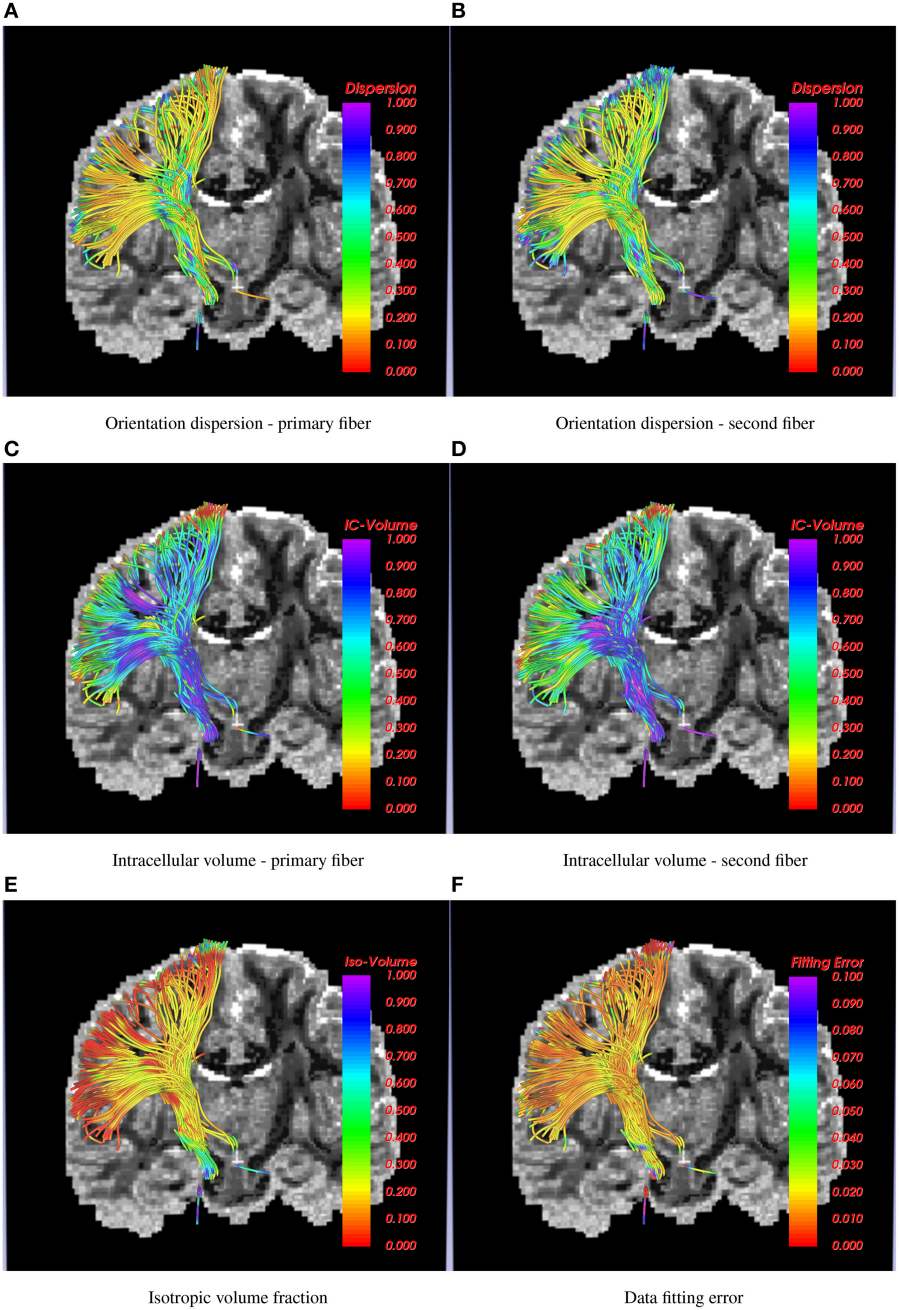
**Corticospinal tract traced using the 2-fiber NODDI model**. In the background is a slice of orientation dispersion obtained from 1-fiber NODDI model of Daducci et al. (2015). Several NODDI specific measures are shown along the tracts along with the data fitting error, which is below 3% in most cases.

Results shown in Figure 4 demonstrate how the proposed method can be used to trace the CST to the hand and face motor areas. The estimated model parameters, such as intracellular volume fraction (for both the fibers of the 2-fiber NODDI model), orientation dispersion and the isotropic volume fraction are also shown. Note once again, that we show the orientation dispersion and intracellular volume fraction for the second fiber as well, although the tracts were obtained by following the primary fibers. The results demonstrate the smooth and robust estimation of all the model parameters, including the ones for the second fiber.

**Figure 5:**
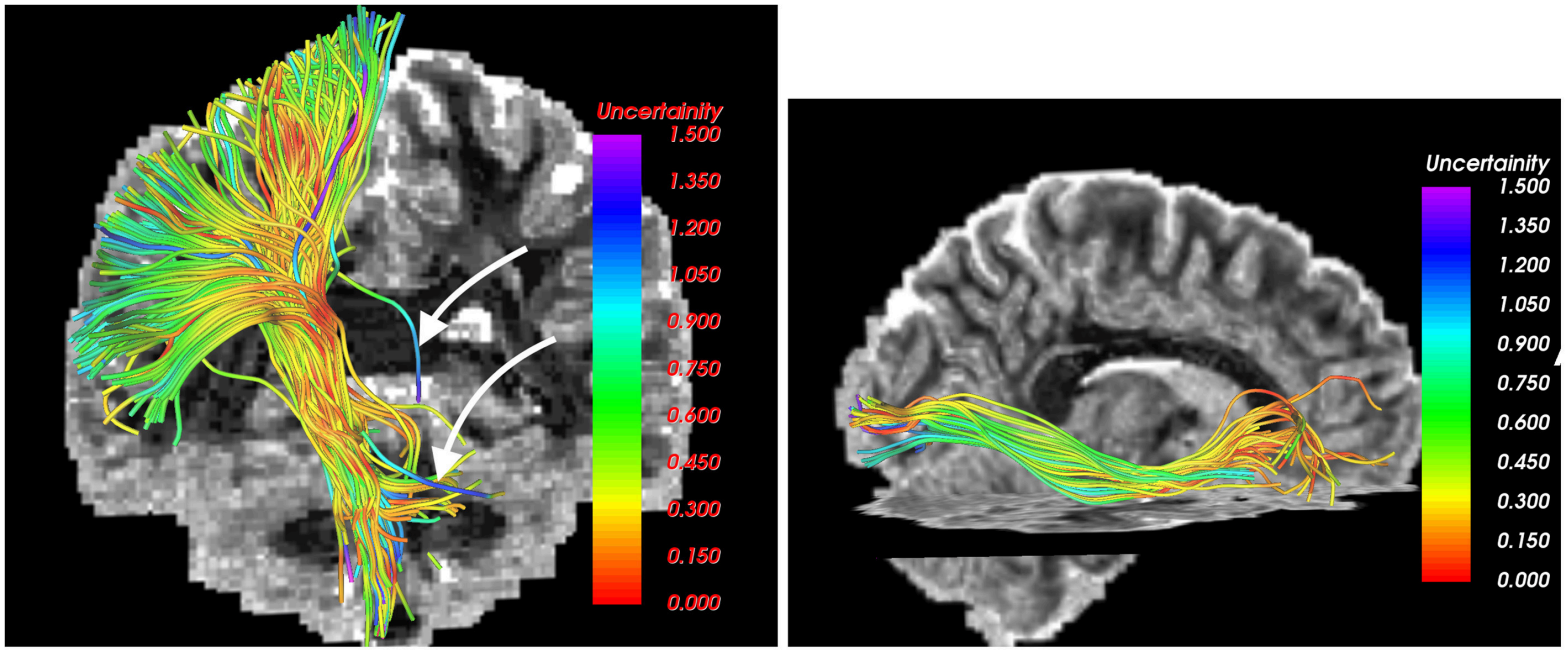
**Estimated uncertainty in the model parameters in the CST (left) and IOFF (right) fiber bundles**. Fibers with high uncertainty (likely false positives), as marked by white arrows, can be easily removed by thresholding the estimated uncertainty.

The UIF filter can also estimate the uncertainty in the estimation of the model parameters. This information can be used to removed unlikely fibers or false positives from the tractography, which offers a powerful way to automatically detect such fibers and remove them. One such example is shown in Figure 5 as pointed by the white arrows. Note that, to the best of our knowledge, only the UIF (and UKF) based tractography methods allow an inherent way to detect unlikely fibers.

**Figure 6:**
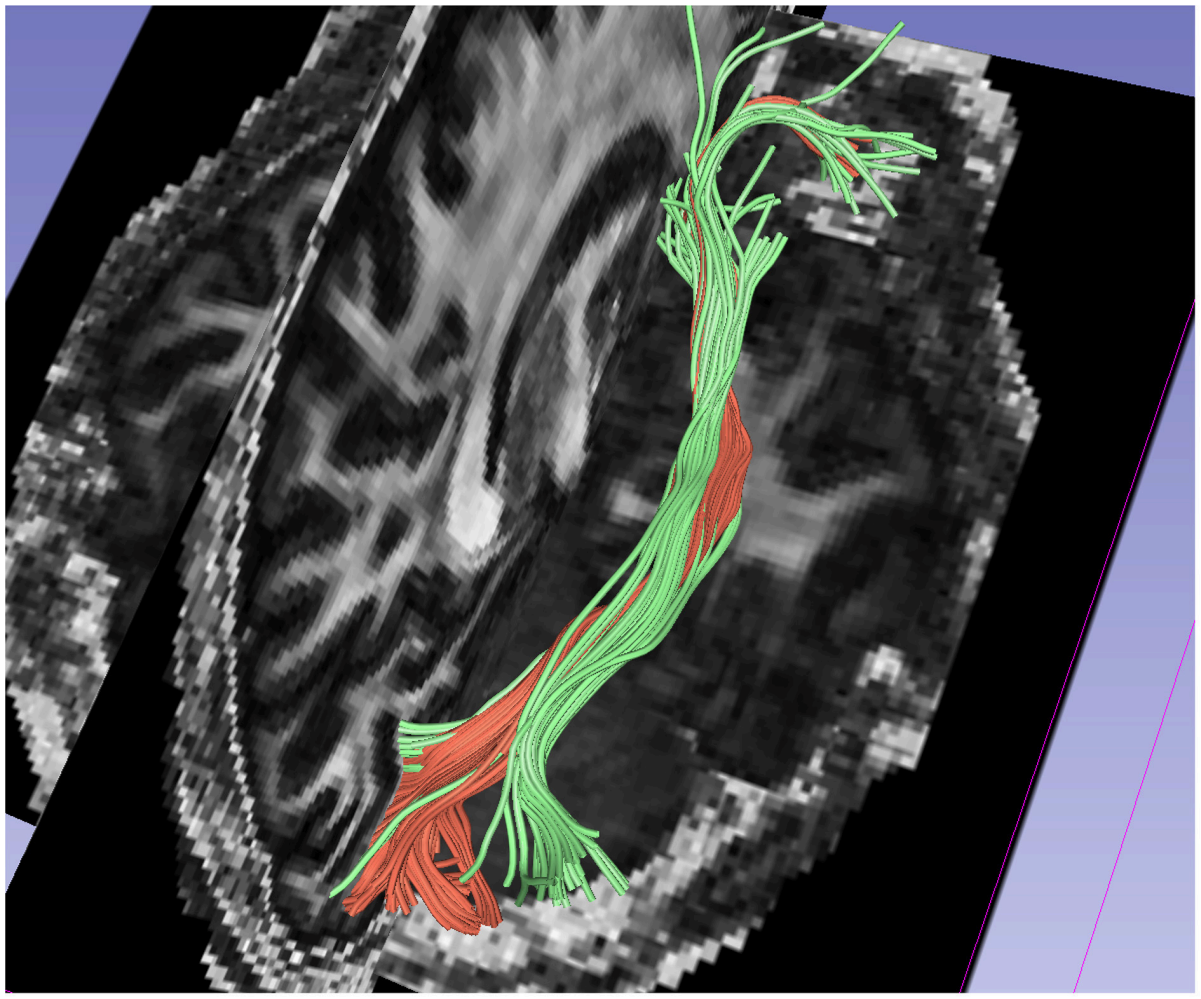
**Comparision of results between 1-fiber NODDI model tractography(red) and 2-fiber NODDI model tractography(green) for the IOFF fibers**.

Figure 6 shows a comparison of the traced inferior occipito-frontal fibers (IOFF) as traced by the 1-fiber (red) and 2-fiber (green) NODDI model. The 2-fiber NODDI manages to trace and connect a different part of gray matter region that is missed by the 1-fiber NODDI model. In fact the 1-fiber tracts only trace the medial portion of the lateral occipital cortex, whereas the 2-fiber NODDI tracts cover most of the lateral-occipital cortex as labeled by Freesurfer. Thus, the 2-fiber NODDI potentially provides a better estimate of fiber connectivity.

## 4. Discussion and limitations

In this work, we applied a new computationally efficient and numerically robust unscented information filtering framework for joint estimation of NODDI parameters and tractography. The proposed UIF filter provides a 2-fold improvement in processing speed, while computing the uncertainty in the estimated parameters, which is generally lacking in most existing methods. The method allows for tracing crossing fibers while accounting for the correlation in diffusion along the tracts. To the best of our knowledge, this is the first time, multi-fiber NODDI model has been used to estimate fiber dispersion index and intracellular-extracellular volume fractions for each of the crossing fibers separately, within a tractography framework. This can be quite useful in understanding the geometric properties of the white matter as it is traced along the tracts. The proposed algorithm is an open-source software and can be downloaded from https://github.com/pnlbwh/ukftractography, with the option of using the NODDI model as one of the possible choices.

We should however note that, the fiber dispersion dispersion (OD) index computed using the NODDI model is different than the one obtained using the methods in Savadjiev et al. (2010, 2012). In particular, the latter are computed from the fiber tracts or tensor fields at a macroscopic level, whereas the fiber dispersion obtained from NODDI is inherently microscopic. While there could be regions where they agree (some regions of white matter), yet in the gray matter the OD measures from NODDI is very different than the fiber dispersion computed using the method in Savadjiev et al. (2012).

**Figure 7:**
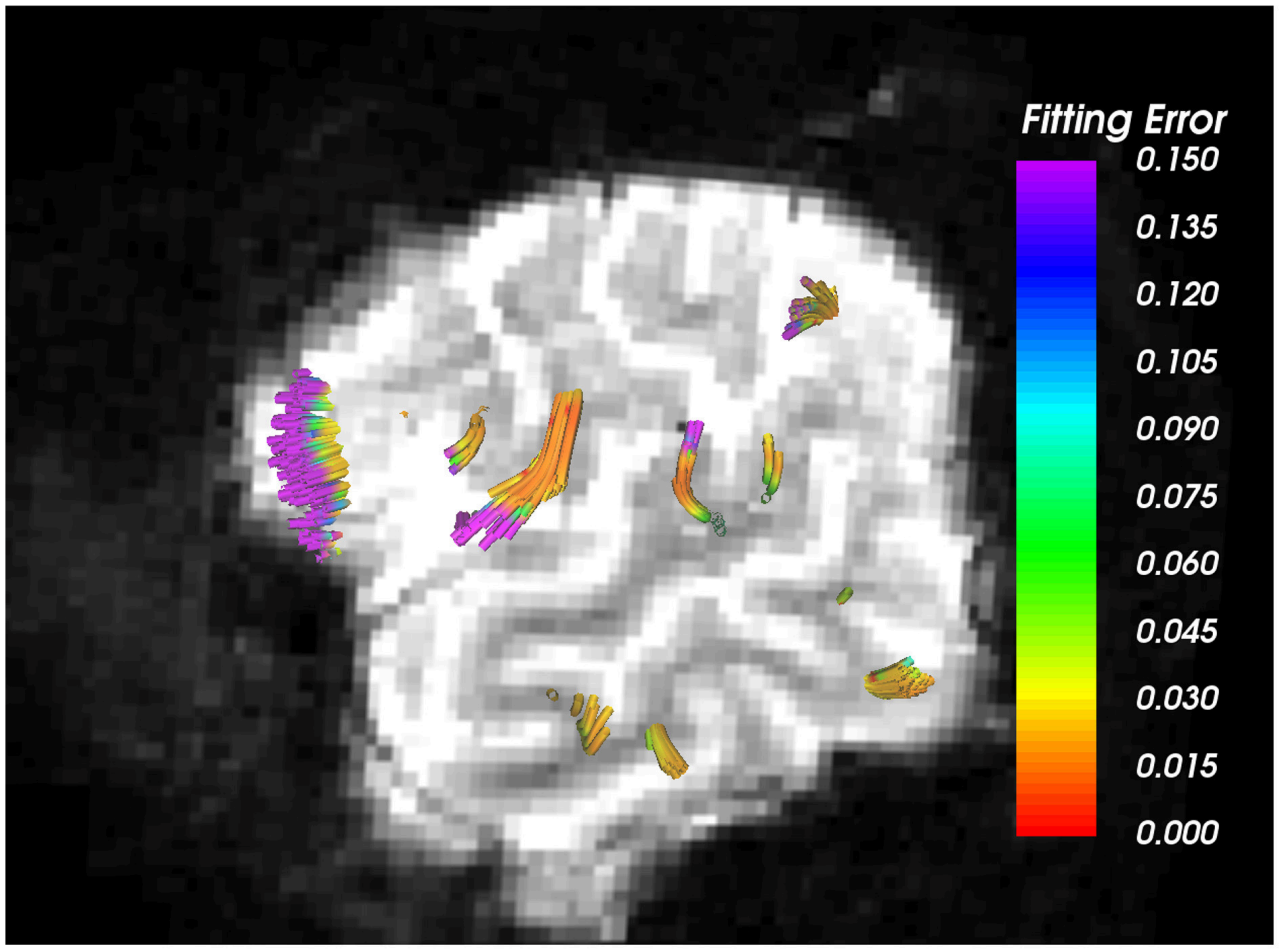
**Arcuate Fasciculus traced using 1-fiber NODDI model**. Background is the *b* = 0 image, where very bright regions indicate CSF areas. As seen, higher error in data fitting occurs only in the CSF areas, which is extremely noisy with free isotropic diffusion.

Nevertheless, the proposed method has a few limitations. First, we assume equal volume fraction for each of the crossing fibers in our 2-fiber NODDI model, which may not be accurate. Second, the model fit to the data in the CSF areas is poor due to high noise in the data itself, as seen in Figure 7. However, the error in most white and gray matter areas is quite low, i.e., less than 2%. Another limitation of the current implementation of the proposed method is its inability to trace more than 2 fiber crossings. While an extension to trace 3 fiber model is straightforward, it can be done in areas which are a-priori known to have 3 fiber crossings. However, we believe that using a 3-fiber model for tracing all fibers would result in over-fitting of the data. Yet, we should note that the a majority of the white matter voxels have two crossings, and a very small region has more than 2 fiber crossings. Thus, the proposed method can be applied in most parts of the brain to trace fibers and estimate the dispersion index of each fiber separately, which is a significant improvement over the existing single-fiber based model of Zhang et al. (2012).

Another limitation, as is the case with most tractography algorithms, is the directional nature of the estimated tracts. For example, the tracts seeded in region A and reaching B, may not be obtained if seeding was done in region B. However, a typical way this particular problem is addressed is by seeding the whole brain and extracting tracts of interest from the whole brain tractography as has been done here and in most works using deterministic tractography.

Nevertheless, we believe that the present method allows to estimate parameters of the NODDI model along fiber tracts and allows to trace fibers through crossing regions. This could be useful in neuroscience studies to detect changes in white matter structure due to disease.

## Author contributions

YR: Conception, Design, Manuscript writing and data analysis. PR: Implementation, paper writing, data analysis.

### Conflict of interest statement

The authors declare that the research was conducted in the absence of any commercial or financial relationships that could be construed as a potential conflict of interest.

## Appendix

### The unscented information filter (UIF)

The state **x**_*i*_ of the UIF is given the unknown parameters of the single or multi-fiber NODDI model described in Equation (3). The initial state **x**_0_ is obtained using a coarse grid search for the unknown parameters as described in Zhang et al. (2012). The initial covariance matrix $P_{0}\in {\mathbb{R}}^{n\times n}$ is assumed to be diagonal with all entries initially set to 0.01. Here *n* is the dimension of the state. There are a total of 2*n*+1 sigma points, with weights $w_{0}=\frac{k}{\left(n+k\right)}$, $w_{i}=\frac{1}{2\left(n+k\right)}$, and ζ = *n*+*k*. *k* is an adjustable scaling parameter set to 0.01 in all our experiments (Wan and Van Der Merwe, 2000).

1. Form weighted sigma points ${\text{X}}_{t}={\left\{w_{i},{\text{x}}_{i}\right\}}_{i=0}^{2n}$ around current mean **x**_*t*_ and covariance *P*_*t*_ with scaling factor ζ
  (A1) $$\begin{array}{l} {\text{X}}_{0}={\text{x}}_{t}\;{\text{X}}_{i}={\text{x}}_{t}+{\left[\sqrt{\zeta P_{t}}\right]}_{i}\;{\text{X}}_{i+n}={\text{x}}_{t}-{\left[\sqrt{\zeta P_{t}}\right]}_{i} \end{array}$$
2. Prediction Equations
  (A2) $$\begin{array}{r} {\text{X}}_{t+1|t}^{x}=s\left[{\text{X}}_{t}\right] \end{array}$$
  (A3) $$\begin{array}{r} {\bar{\text{x}}}_{t+1|t}=\underset{i}{\sum }w_{i}\;{\text{X}}_{i,t+1|t}^{x} \end{array}$$
  Predicted State covariance matrix
  (A4) $$\begin{array}{l} P_{t+1|t}=\underset{i}{\sum }w_{i}\left({\text{X}}_{i,t+1|t}^{x}-{\bar{\text{x}}}_{t+1|t}\right){\left({\text{X}}_{i,t+1|t}^{x}-{\bar{\text{x}}}_{t+1|t}\right)}^{T} \end{array}$$
  Information prediction equations
  (A5) $$\begin{array}{r} {\text{Y}}_{t+1|t}={\left(P_{t+1|t}\right)}^{-1} \end{array}$$
  (A6) $$\begin{array}{r} {\bar{\text{y}}}_{t+1|t}={\text{Y}}_{t+1|t}{\bar{\text{x}}}_{t+1|t} \end{array}$$
3. Predict new sigma points for update, e.g.,
  (A7) $$\begin{array}{rcl} {\text{X}}_{0,t+1|t} & = & {\bar{\text{x}}}_{t+1|t}\;{\text{X}}_{i,t+1|t}={\bar{\text{x}}}_{t+1|t}+{\left[\sqrt{\zeta P_{t+1|t}}\right]}_{i}\\ {\text{X}}_{i+n,t+1|t} & = & {\bar{\text{x}}}_{t+1|t}-{\left[\sqrt{\zeta P_{t+1|t}}\right]}_{i} \end{array}$$
4. Measurement update equations for **y**_*t*_ are
  (A8) $$\begin{array}{rcl} {\text{Z}}_{t+1|t} & = & h\left[{\text{X}}_{t+1|t}\right] \end{array}$$
  (A9) $$\begin{array}{rcl} {\bar{\text{z}}}_{t+1|t} & = & \underset{i}{\sum }w_{i}\;{\text{X}}_{i,t+1|t} \end{array}$$
  (A10) $$\begin{array}{rcl} P_{xz} & = & \underset{i}{\sum }w_{i}\left({\text{X}}_{i,t+1|t}-{\bar{\text{x}}}_{t+1|t}\right){\left({\text{Z}}_{i,t+1|t}-{\bar{\text{z}}}_{t+1|t}\right)}^{T} \end{array}$$
  (A11) $$\begin{array}{rcl} H_{t+1} & = & {\text{Y}}_{t+1|t}P_{xz} \end{array}$$
  (A12) $$\begin{array}{rcl} i_{t+1} & = & H_{t+1}^{T}R_{t+1}^{-1}H_{t+1} \end{array}$$
  (A13) $$\begin{array}{rcl} {\text{I}}_{t+1} & = & H_{t+1}^{T}R_{t+1}^{-1}\left(z-{\text{z}}_{t+1|t}+{\left(P_{xz}\right)}^{T}{\bar{\text{y}}}_{t+1|t}\right) \end{array}$$
  (A14) $$\begin{array}{rcl} P_{t+1} & = & {\left({\text{Y}}_{t+1|t}+{\text{I}}_{t+1}\right)}^{-1}\\ {\text{X}}_{t+1} & = & P_{t+1}\left(i_{t+1}+{\bar{\text{y}}}_{t+1|t}\right) \end{array}$$

Note that, the matrix **I**_*t*_ above, is the Fisher Information matrix and can be potentially used to determine the amount of information that the observed measurement *z* carries about each of the model parameters. In the above formulation, we have ignored the constraints on our model. This could result in non-realistic estimates, i.e., the volume fractions may not lie between 0 and 1. To enforce appropriate constraints, one can directly project any unconstrained state **x** onto the constrained subspace as shown in Simon and Simon (2006). In other words, we wish to find the state $\hat{\text{x}}$ closest to the unconstrained state **x** which still obeys the constraints, $C\hat{\text{x}}\leq \text{d}$. For example, for the single-fiber NODDI model, the state is $\text{x}=\left[V_{ic}\kappa \text{m}V_{iso}\right]\in {\mathbb{R}}^{6\times 1}$. In this case, one can define the matrix *C* and *d* by:

(A15) $$\begin{array}{l} C=\left[\begin{array}{cccc} 1 & 0 & 0 & 0\\ -1 & 0 & 0 & 0\\ 0 & -1 & 0 & 0\\ 0 & 0 & 0 & 1\\ 0 & 0 & 0 & -1 \end{array}\right],\;d=\left[\begin{array}{c} 1\\ 0\\ 0\\ 1\\ 0 \end{array}\right]. \end{array}$$

Using *P*_*t*_ as a weighting matrix, this becomes a quadratic programming problem:

(A16) $$\begin{array}{l} \underset{\hat{\text{x}}}{\min}\;{\left(\text{x}-\hat{\text{x}}\right)}^{T}P_{t}^{-1}\left(\text{x}-\hat{\text{x}}\right)\text{ subject to }C\hat{\text{x}}\leq \text{d}. \end{array}$$

This projection procedure is applied within the UIF algorithm to correct at every place where we move in the state-space: after spreading the sigma points **X**_*t*_, after transforming the sigma points **X**_*t*+1|*t*_, and after the final estimate **x**_*t*+1_.
